# Non-steroidal anti-inflammatory drug-induced enteropathy as a major risk factor for small bowel bleeding: a retrospective study

**DOI:** 10.1186/s12876-020-01329-5

**Published:** 2020-06-08

**Authors:** Doo-Ho Lim, Kyoungwon Jung, Seung Bum Lee, In Kyu Park, Hee Jeong Cha, Jae Ho Park, Byung Gyu Kim, Seok Won Jung, In Du Jeong, Jae Hyun Kim, Sung Eun Kim, Won Moon, Moo In Park, Seun Ja Park

**Affiliations:** 1 Department of Internal Medicine, University of Ulsan College of Medicine, Ulsan University Hospital, 877 Bangeojinsunhwando-ro, Dong-gu, Ulsan, 44033 South Korea; 2 Department of Internal Medicine, Kosin University College of Medicine, Kosin University Gospel Hospital, Busan, South Korea; 3 Department of General Surgery, University of Ulsan College of Medicine, Ulsan University Hospital, Ulsan, South Korea; 4 Department of Pathology, University of Ulsan College of Medicine, Ulsan University Hospital, Ulsan, South Korea

**Keywords:** Capsule endoscopy, Nonsteroidal anti-inflammatory drugs, Obscure gastrointestinal bleeding, Iron deficiency anemia, Angioectasia

## Abstract

**Background:**

Small bowel (SB) bleeding accounts for 5% of all gastrointestinal (GI) bleeding cases and 80% of obscure GI bleeding cases. Although angioectasia is the common etiology of SB bleeding, nonsteroidal anti-inflammatory drug (NSAID)-induced SB lesions are also reported as a major cause in studies from Eastern countries. Herein, we assessed the frequency of occurrence of NSAID-induced SB lesions in Korean patients with obscure GI bleeding.

**Methods:**

We retrospectively analyzed medical records of all consecutive patients aged ≥18 years who underwent capsule endoscopy from March 2018 to February 2019 at Ulsan University Hospital and Kosin University Gospel Hospital.

**Results:**

Of the 83 subjects (all Korean; mean age ± standard deviation: 59 ± 18 years; age range: 18–84 years; men: *n* = 52; women: *n* = 31), 55 (66.2%) had stool with clear blood and 28 (33.8%) had normal stool with iron deficiency anemia. The detection rate of SB bleeding and lesions using capsule endoscopy was 72.3% (60 of 83 patients). A significantly higher frequency (40 of 51) of ulcerative/erosive lesions than other causes was observed in patients with inactive bleeding but visible SB lesions. As a result, NSAID-induced enteropathy accounted for 30.1% of 83 patients with obscure GI bleeding (25 of the all 60 SB bleeding cases).

**Conclusions:**

Contrary to what is reported for patients in Western countries, this study in Korean patients showed an improved diagnostic yield of capsule endoscopy for obscure GI bleeding and that NSAID-induced enteropathy was the most common etiology of SB bleeding. Aggressive small intestine examination is required for patients with unexplained GI bleeding.

## Background

Obscure gastrointestinal (GI) bleeding is defined as persistent or recurrent bleeding of unknown origin despite repetitive diagnostic testing, including upper GI endoscopy and colonoscopy [1]. Small bowel (SB) bleeding accounts for 80% of obscure GI bleeding cases and 5% of all GI bleeding cases. Unexplained iron deficiency anemia (IDA) manifests in 30–40% of obscure GI bleeding cases [2]. Capsule endoscopy is the first-line examination in cases of obscure GI bleeding and the third diagnostic test after negative upper and lower endoscopy results in cases of ongoing overt bleeding [3–6]. Whereas angioectasia is the most common etiology of SB bleeding in Western countries [4, 7, 8], nonsteroidal anti-inflammatory drug (NSAID)-induced SB lesions are the major cause reported in Japanese studies [9, 10].

NSAIDs are widely prescribed in most clinical conditions; however, it is well known that these drugs can cause GI complications [11]. Although various studies have reported an association between NSAID intake and GI adverse effects [12, 13], the role of NSAIDs in SB bleeding remains to be elucidated. Therefore, we aimed to assess the frequency of NSAID-induced SB lesions in Korean patients with obscure GI bleeding, who underwent capsule endoscopy.

## Methods

### Study population

We retrospectively evaluated the medical records of all consecutive patients aged ≥18 years with obscure GI bleeding who underwent capsule endoscopy from March 2018 to February 2019 at Ulsan University Hospital and Kosin University Gospel Hospital. The indication for the test included bloody stool in patients with an unidentified source of bleeding and lesions during diagnostic evaluations, including upper GI endoscopy, colonoscopy, and abdominal computed tomography (CT) in the last 3 months [5]. We also included patients who had no visible bloody stool, showed persistent or repeated exacerbation of IDA despite iron supplementation for > 6 months, and had no hemorrhagic lesions identified during the above screening methods in the last 3 months [14]. If CT-scan had been performed in the last 1 year, in some patients, the test was not performed again when anemia was confirmed. Rebleeding was defined as recurrent anemia (≥2 g/dL decrease in the hemoglobin level), overt melena/ hematochezia, or occult GI bleeding during the follow-up period [15]. This study was approved by the Institutional Review Boards (IRB of the Ulsan University Hospital (IRB No. 2019–10-014) and the Kosin University Gospel Hospital (IRB No. 2019–10-001)). The requirement for informed consent from patients was waived because patient records and information were de-identified prior to analysis.

### Capsule endoscopy

Capsule endoscopy was performed using Pillcam® (SB3, Given Imaging Ltd., Yoqneam, Israel) and MiroCam® (MC1200, IntroMedic Ltd., Seoul, Korea) devices. Given that both pieces of equipment have 12 h of battery life, all endoscopies were performed for 12 h. All patients fasted for 12 h and received 40 mg of oral simethicone before the procedure to prevent air bubbles from forming [16]. Moreover, we used 2 L polyethylene glycol solution at least 2–16 h before examination to improve the quality of the SB image [17, 18]. Patients with bloody stool were examined in the hospital in all cases; based on the time at which the blood stool was noted, the capsule endoscope was able to start testing within a median of 51 h (inter-quartile range, 24 to 96 h). Those who were referred due to anemia had an outpatient examination if there was no bloody stool. To prevent capsule retention, we checked the presence of small bowel stenosis on the CT scan that was performed at least 1 year before the capsule endoscopy. Five gastroenterologists with extensive experience in GI endoscopy reviewed and extensively discussed all capsule video images to reach a diagnosis. An erosion was defined as a roundish area of mucosal disruption smaller than a diameter equivalent to 1 circular fold of the mucosa (also called the valves of Kerckring, valvulae conniventes, or plicae circulares) [19]. An ulcerative lesion was defined as a mucosal penetration with a diameter larger than 1 circular fold of the mucosa [15]. The etiology of ulcerated lesions was determined based on clinical information and endoscopic findings. We examined NSAID-induced enteropathy based on the following criteria: history of NSAID use within the previous 1 month; endoscopic findings, including ulcers, erosions, scar changes, or luminal stenosis; improvement of the clinical course and/or endoscopic findings after cessation of NSAID use; and exclusion of other etiologies, including infection, inflammatory bowel disease, or malignancy [12, 20, 21]. There is no criterion regarding the duration of NSAID use to diagnose NSAID-induced enteropathy. However, because SB injuries were observed in 68% of healthy volunteers taking NSAIDs for only 1–2 weeks [20, 22], we chose to include patients who had history of NSAID use within the previous 1 month, as was done in a previous study [21].

### Statistical analysis

Continuous variables were compared using Student’s *t*-test and categorical variables were analyzed with a chi-square or Fisher’s exact test. A two-tailed *p*-value < 0.05 was considered statistically significant. All statistical analyses were performed using the SPSS statistical package for Windows, Version 24.0 (SPSS Inc., Chicago, IL, USA).

## Results

### Sample analysis

**Fig. 1:**
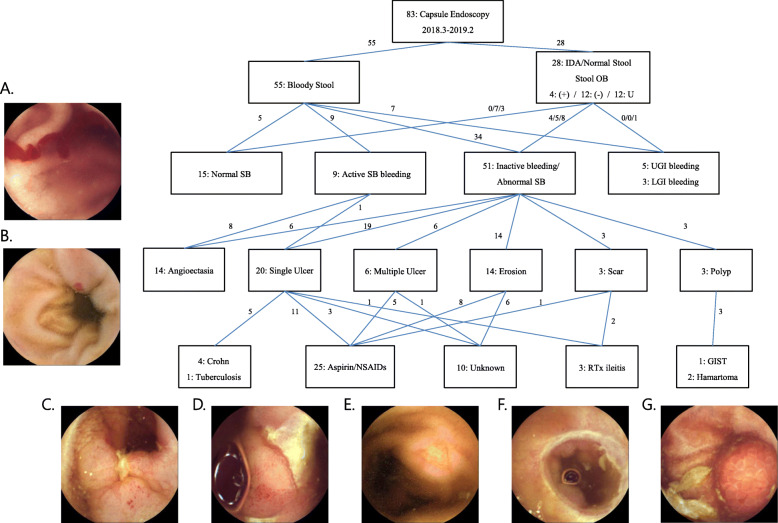
Representative capsule endoscopy images of small bowel lesions. **a**. Angioectasia with active bleeding. **b**. Angioectasia with no bleeding. **c**. Ulcer suggestive of Crohn’s disease. **d**. Ulcer suggestive of NSAID-induced enteropathy. **e**. Erosion suggestive of NSAID-induced enteropathy. **f**. Radiation ileitis. **g**. SB polyp (GIST). IDA, iron deficiency anemia; OB, occult blood; SB, small bowel; UGI, upper gastrointestinal; LGI, lower gastrointestinal; NSAIDs, nonsteroidal anti-inflammatory drugs; RTx, radiotherapy; GIST, gastrointestinal stromal tumor

We assessed 83 subjects (52 men and 31 women) who underwent capsule endoscopy during the study period (see Table 1 for patient characteristics). The subjects were Korean and aged 18–84 years (mean ± standard deviation: 59 ± 18 years). Of these, 55 (66.2%) patients had clear bloody stool and 28 (33.8%) had normal stool; all had IDA (Fig. 1). The mean small bowel transit time was 6.39 h (range, 2.42–11.38 h). Capsule retention did not occur in this study. Most patients (92.4%) showed scores of 3 or 2 on the Boston bowel preparation scale [23]. A total of 37 (44.6%) patients had a history of taking NSAIDs/low-dose aspirin (Table 2). The brand name and dosage of NSAIDs were not clearly identified in the medical records. Musculoskeletal disorders were the most common indication for taking NSAIDs, while coronary artery disease and other cardiovascular diseases were the main indications for taking aspirin. The duration of NSAID use varied from 2 weeks to 10 years prior to the capsule endoscopy. An association with SB bleeding was considered only when NSAIDs/low-dose aspirin was taken within the previous 1 month. Of the 37 patients, 33 had SB lesions; of these, 25 patients were diagnosed with NSAID-induced enteropathy without any other cause of SB bleeding. The main treatments for NSAID-induced enteropathy were discontinuation of NSAIDs or the replacement of NSAIDs with drugs with a low risk of bleeding. Among 37 patients with a history of low-dose aspirin or NSAID medication, only 3 who were treated with selective cyclooxygenase (COX)-2 inhibitors had normal stool. Most patients with active bleeding during endoscopy had angioectasia (8 of 9 patients). A fecal occult blood test was performed in patients with normal stool (16 of 28) and the results were negative in 12 of these patients, among which 7 had no SB lesions. A significantly higher frequency (40 of 51) of ulcerative/erosive lesions was observed in patients with inactive bleeding but visible SB lesions than other causes; among these patients, 25 had a history of low-dose aspirin or NSAID medication. As a result, NSAID-induced enteropathy accounted for 30.1% of 83 patients with obscure GI bleeding (25 of all 60 SB bleeding cases). Four cases of previously undiagnosed Crohn’s disease and 1 case of tuberculosis were identified. Other uncommon etiologies associated with unknown GI bleeding included radiation ileitis (*n* = 3) and SB polyps (*n* = 3).

**Table 1:** Clinical characteristics of patients

Characteristic	Overall (*n* = 83)	Bloody stool (*n* = 55)	Normal stool (*n* = 28)	*p*-value
Age, yr.	59 ± 18	61 ± 19	56 ± 15	0.185
Sex, male	52 (62.7)	37 (67.3)	15 (53.6)	0.222
Aspirin/NSAID	37 (44.6)	25 (45.5)	12 (42.9)	0.822
Hemoglobin (g/dL)	8.7 ± 3.0	8.7 ± 2.8	8.9 ± 3.5	0.785
Albumin (g/dL)	3.8 ± 0.6	3.7 ± 0.7	3.9 ± 0.6	0.191
Small bowel bleeding	60 (72.3)	43 (78.2)	17 (60.7)	0.093
Specific lesion^a^				
Angioectasia	14 (23.3)	14 (32.6)	0 (0.0)	
NSAID-induced	25 (41.7)	15 (34.9)	10 (58.8)	0.024
enteropathy	21 (35.0)	14 (32.6)	7 (41.2)	
Other causes^b^				
Site of bleeding^a^				
Jejunum	16 (26.7)	14 (36.2)	2 (11.8)	
Ileum	38 (63.3)	26 (60.5)	12 (70.6)	0.169
Indeterminate	6 (10.0)	3 (7.0)	3 (17.6)	

^a^The denominator is 60 patients with confirmed small bowel lesion

^b^Other causes of small bowel bleeding, including Crohn’s disease, intestinal tuberculosis, radiation ileitis, small bowel polyps, and small bowel lesions with unknown causes

Categorical and continuous variables are presented as number (%) and mean ± SD, respectively

*SD* Standard deviation; *NSAID* Nonsteroidal anti-inflammatory drug; *Hb* Hemoglobin

**Table 2:** Details of NSAIDs/low-dose aspirin

	*N* = 37 (100%) (NSAID = 17/Low-dose aspirin = 21)^a^
Indication	
Coronary artery disease	11 (29.7)
Other cardiovascular disease	9 (24.3)
Musculoskeletal disease	15 (40.5)
Unknown	3 (8.1)
Duration	
< 1 month	2 (5.4)
1–6 months	11 (29.7)
> 6 months	22 (59.5)
Unknown	2 (5.4)
Concurrent medication	
Clopidogrel	6 (16.2)
Warfarin/NOAC	7 (18.9)
Other anti-platelet agent	3 (8.1)
None	21 (56.8)
Site of bleeding	
Small bowel	33 (89.2)
Stomach	2 (5.4)
Undetermined	2 (5.4)
Etiology of small bowel bleeding^b^	
NSAID-induced enteropathy	25 (75.8)
Angioectasia	7 (21.2)
Others	1 (3.0)
Initial management of NSAID-induced enteropathy^c^	
Discontinue NSAID/low-dose aspirin	20 (80.0)
Continue NSAID/low-dose aspirin	2 (8.0)
+ Mucosal protective agent^d^	
Unknown	3 (12.0)

^a^ One patient had a history of both NSAID and low dose aspirin

^b^ The denominator is 33 patients with confirmed small bowel bleeding on capsule endoscopy

^c^ The denominator is 25 patients with small bowel bleeding associated with NSAID/low-dose aspirin

^d^ Mucosal protective agent indicates rebamipide, sucralfate, or sodium alginate

NSAID, nonsteroidal anti-inflammatory drug; NOAC, non-vitamin K antagonist oral anticoagulant

### Evaluation of rebleeding and treatment

**Fig. 2:**
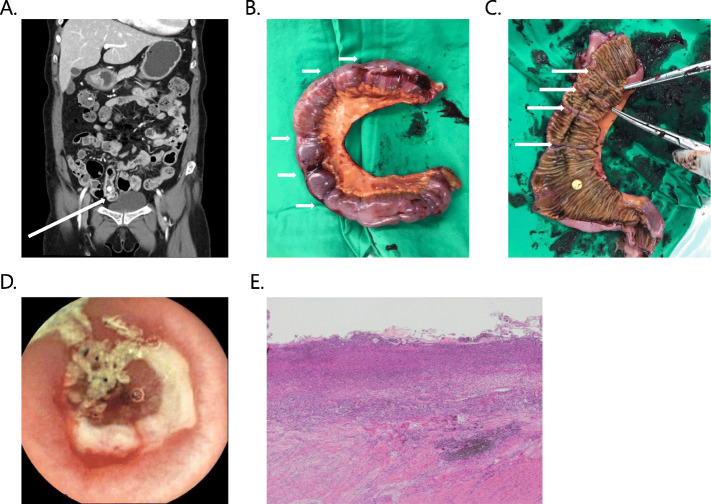
Surgical intervention in a patient with small-bowel stricture. **a**. Abdominal computed tomography showing a stricture in the distal ileum. **b**. Gross findings after bowel resection with multiple stricture sites. **c**. Surgical resection exposing the inside of the small bowel. **d**. Capsule endoscopy image showing a semi-circular ulcer with active hemorrhage. **e**. Histologic findings show ulceration including diffuse loss of villi, mucosal and submucosal neutrophilic exudates, and transmural inflammation (Hematoxylin and Eosin × 40)

Prior to capsule endoscopy, 58 (69.9%) patients had anemia with hemoglobin < 10 g/dL. SB bleeding was predominant in > 50% (17 of 28) of patients with normal stool. All patients with SB bleeding due to angioectasia showed bloody stool, while ulcerative lesions were prevalent in patients with normal stool. Rebleeding occurred in 7 patients during the 12-month follow-up period (Table 3). Two patients with Crohn’s disease who presented with persistent IDA required specific treatments: One patient was given thiopurine, but the anemia continued and so thiopurine was replaced with methotrexate; no recurrence of anemia has been reported since then. In the other case, administration of immunosuppressive drugs, including thiopurine, failed continuously, mainly due to leukocytopenia associated with persistent IDA; this patient underwent resection of a short segment of the small bowel in which ulcers were observed. After the surgery, anemia has not recurred so far. One patient who was considered to have Crohn’s disease initially, presented with multiple lymphadenopathy during the follow-up period and was finally diagnosed with tuberculosis. Among 2 patients with radiation ileitis, 1 underwent surgical treatment due to recurrent life-threatening bloody stool, whereas in the other patient with persistent IDA, medical treatment was continued with prolonged oral iron replacement. A patient with a rebleeding episode from angioectasia received surgery. Lastly, one patient who received NSAIDs to treat fibromyalgia for > 2 years presented with an ulcerative lesion, had unresolved anemia, and showed recurrent abdominal pain, which lasted for > 6 months, despite adequate iron administration and stopping NSAIDs. Capsule endoscopy showed multiple small bowel ulcers and an additional abdominal CT at the recurrence of bleeding revealed multiple strictures of the SB, which was not detected previous CT-scan performed 8 months ago; eventually segmental resection of the SB was performed, through which 5 sites of stenosis in the distal ileum were identified (Fig. 2). No recurrence of anemia or abdominal pain was present 6 months postoperatively.

**Table 3:** Rebleeding cases

No.	Indication for capsule endoscopy	Cause of small bowel bleeding	Final diagnosis	Timing of rebleeding after diagnosis	Specific therapy	Treatment outcome
1	Bloody stool	Ulcer	Crohn’s disease	23 days	Thiopurine	Persistent IDA^c^
2	Bloody stool	Diffuse scar change	Radiation ileitis^a^	87 days	Surgery	No further bleeding
3	Bloody stool	Ulcer	Crohn’s disease	117 days	5-aminosalicylic acid	Persistent IDA^c^
4	Bloody stool	Ulcer with stricture	Radiation ileitis^a^	49 days	Iron replacement	Follow-up loss
5	IDA	Ulcer	Intestinal Tbc^b^	146 days	Anti-Tbc medication	Recovered IDA
6	IDA	Ulcer with stricture	NSAID-induced enteropathy	15 days	Surgery	Recovered IDA
7	Bloody stool	Angioectasia	Angioectasia	330 days	Surgery	No further bleeding

^a^ Indication for radiation therapy was uterine cervical cancer

^b^ The patient who was considered as having Crohn’s disease initially, presented with multiple lymphadenopathy and was finally diagnosed with tuberculosis

^c^Patient No.1 was given methotrexate instead of thiopurine and no recurrence of anemia has been reported since then; Patient No.3 underwent resection of a short segment of the small bowel and anemia has not recurred so far

*IDA* Iron deficiency anemia; *Tbc* Tuberculosis; *NSAID* Nonsteroidal anti-inflammatory drug

## Discussion

Capsule endoscopy for unexplained GI bleeding has shown diagnostic yields of 57–62% [24], with the most common diagnosis being angioectasia (50%), followed by ulcers (26.8%) and tumors (8.8%), as suggested in a recent systematic literature review [14]. The present study revealed a detection rate of SB bleeding and lesions using capsule endoscopy of 72.3% (60 of 83 patients), of which NSAID-induced enteropathy accounted for 25 of all 60 SB bleeding cases. The diagnostic yield of small bowel lesions in our study was higher than in a previous nationwide Korean study of capsule endoscopy [25], but the prevalence of ulcerative/erosive lesions was comparable (40/60 vs 106/157). Similar to our results, a Korean study using balloon-assisted enteroscopy found that the most common type of SB lesions associated with obscure GI bleeding were mucosal injury (56%), followed by vascular lesions (18.7%) [26]. Because of limited availability of data regarding the use of NSAIDs/low-dose aspirin, we could not compare the prevalence of NSAID-induced enteropathy with the results from these studies. However, considering the meaningful association between NSAID use and SB injury presented in previous studies [22, 27], NSAID-induced enteropathy could be the major cause of obscure GI bleeding in Eastern countries. Contrary to previous reports from Western countries, we observed a higher occurrence rate of ulcerative/erosive lesions than angioectasia in patients with obscure GI bleeding, supporting the implication of low-dose aspirin or NSAID medications in the disease etiology. A recent systematic review and meta-analysis suggested that the optical timing of capsule endoscopy would be within 2 days, to improve the diagnostic yield [28]. For patients with clear bloody stool in our study, the capsule endoscope was able to start testing within a median of 51 h (inter-quartile range, 24 to 96 h). In the case of persistent melena or hematochezia enough to show symptoms, the patients usually visited the emergency room and in these patients, the capsule endoscopy could be performed within at least 48 h, but if patients showed intermittent blood stool or if symptoms related to bleeding or anemia were not clear, capsule endoscopy would not be conducted within 2 days. In real practice, further improvement is needed in this respect.

NSAIDs are frequently used anti-inflammatory analgesic agents that represent 7.7% of worldwide prescriptions, of which 90% are prescribed to elderly (> 65 years) patients [29]. The mechanism of NSAID-induced enteropathy is supposed to be mediated through COX inhibition [30]. Administration of low-dose aspirin (an irreversible nonselective COX inhibitor) is also associated with SB mucosal injuries; large erosions or ulcers were reported in 60% of healthy volunteers who took 100 mg of low-dose enteric-coated aspirin [31, 32]. In the present study, a history of low-dose aspirin or NSAID use was common in patients with obscure GI bleeding (44.6%), showing a higher frequency of SB ulcerative lesions than of other sources of lesions (angioectasia, *n* = 6; upper or lower GI bleeding, *n* = 6).

Prostaglandins (PG) play an important role in regulating GI blood flow and mucus production; therefore, NSAID-induced suppression of PG production has been implicated in SB damage [33, 34]. Previously, COX-1 inhibition was regarded to be dominantly related to GI mucosal injuries. However, in a recent animal model study, damage to the SB developed only when both COX-1 and COX-2 were inhibited [35]. This result indicates that COX-2-derived PGs also play an important role in the maintenance of tissue integrity and repair of mucosal injury. However, clinical research has shown conflicting results. Several studies have shown an improved GI safety profile with selective COX-2 inhibitors compared to nonselective NSAIDs [36, 37], while others studies indicated no significant differences in SB injuries between these NSAIDs [38, 39]. In the current study, among 37 patients with a history of low-dose aspirin or NSAID medications, the 3 treated with selective COX-2 inhibitors had normal stool, suggesting favorable GI outcomes with selective COX-2 inhibitor therapy. Considering that selective COX-2 inhibitors are not completely safe for the SB, further long-term studies with a larger sample size are warranted to establish the safety profile of the drug in the SB.

Furthermore, the impact of capsule endoscopy on clinical outcomes remains controversial despite reports of SB mucosal damage in 70% of patients taking NSAIDs [22, 40], because it remains unclear whether SB mucosal injuries contribute to significant bleeding [41]. Although patients with NSAID-induced SB injury show low frequency of severe bleeding in the SB [42], rebleeding rates of 21–35% have been reported in patients with SB ulcerations during a mean follow-up period of 17.1–29.7 months [15, 43]. These reports suggest a clinical implication of SB ulcers, which cannot be ignored.

The most effective method of preventing NSAID-induced enteropathy is discontinuation of NSAIDs if possible [12]. Previously, there was no strategy to prevent NSAID-induced enteropathy [13, 44]. However, a recent study reported the effectiveness of misoprostol in the treatment of SB ulcer bleeding associated with aspirin [45]. On the contrary, lesions that induce stenosis, which may not be treated with medication alone, require endoscopic or surgical interventions [46]. In the present study, a patient suffering from fibromyalgia developed SB stricture after NSAID medications for > 2 years and eventually underwent surgical resection.

This study has several limitations. First, it was a retrospective analysis with a small sample size, with insufficient power to detect a significant effect. Second, because balloon-assisted enteroscopy was not routinely performed, pathological findings could not confirm SB ulcers. Third, the short follow-up period prevented the adequate assessment of risk factors for rebleeding. Finally, the fecal occult blood test could not be performed in 12 of 28 patients who presented with normal stool, thereby limiting the interpretation of the results.

## Conclusions

This study showed an improved diagnostic yield of capsule endoscopy for obscure GI bleeding and reaffirmed that NSAID-induced enteropathy is the most common etiology of SB bleeding in Korean patients. Therefore, aggressive clinical management, including SB capsule endoscopy, should be considered for patients with unexplained GI bleeding or drug-refractory iron deficiency anemia, particularly during aspirin or NSAID medications.

## Data Availability

The datasets used and/or analyzed during the current study are available from the corresponding author on request.

## Abbreviations

GI: Gastrointestinal
SB: Small bowel
NSAIDs: Nonsteroidal anti-inflammatory drugs
COX: Cyclooxygenase
PG: Prostaglandin
CT: Computed tomography
